# Optimizing Biologic Therapy for the Prevention of Post-Operative Recurrence in Crohn’s Disease: Current Evidence and Future Perspectives

**DOI:** 10.3390/biomedicines13051232

**Published:** 2025-05-19

**Authors:** Reem Aljabri, Saqer Al-Saraie, Ahmed Alhouti

**Affiliations:** Division of Gastroenterology, Department of Internal Medicine, Ministry of Health of Kuwait—Farwaniya Hospital, Sabah Al-Nasser 92426, Kuwait; lalb5@hotmail.com (S.A.-S.); dr_alhouti@yahoo.com (A.A.)

**Keywords:** Crohn’s disease, post-operative recurrence, biologic therapy, IL-23 inhibitors, JAK inhibitors

## Abstract

Crohn’s disease (CD) imposes a substantial burden on patients due to its chronic, relapsing nature, often necessitating surgical intervention. However, surgery is not curative, and post-operative recurrence (POR) remains a major clinical challenge, with up to 80% of patients developing endoscopic recurrence within one year if left untreated. The pathophysiology of POR is multifactorial, involving dysregulated immune responses, gut microbiota alterations, and mucosal healing impairment, highlighting the need for targeted therapeutic strategies. This review aims to explore the current landscape of POR management, focusing on biologic therapies and emerging advanced treatments. Conventional management relies on early prophylactic therapy with anti-TNF agents such as infliximab and adalimumab, which have demonstrated efficacy in reducing endoscopic and clinical recurrence. However, newer biologics, including IL-23 inhibitors (risankizumab) and Janus kinase (JAK) inhibitors (upadacitinib), have shown promise in CD management, though their role in POR remains underexplored. The lack of direct clinical evidence for advanced biologics in POR prevention, combined with inter-individual variability in treatment response, underscores the need for further research. Future directions should focus on optimizing therapeutic strategies through personalized medicine, identifying predictive biomarkers, and conducting robust trials to establish the efficacy of novel agents in POR prevention. A tailored, evidence-driven approach is essential to improving long-term outcomes and minimizing disease recurrence in post-operative CD patients.

## 1. Introduction

Crohn’s disease (CD) is a chronic inflammatory bowel disease (IBD) characterized by relapsing and remitting symptoms, leading to significant morbidity and a decreased quality of life [1]. The disease can affect any part of the gastrointestinal tract, most commonly the terminal ileum and colon, resulting in symptoms such as abdominal pain, diarrhea, weight loss, and fatigue [2]. The etiology of CD is multifactorial, involving genetic predisposition, environmental factors, and an abnormal immune response [3,4].

Surgical intervention plays a crucial role in the management of CD, particularly when complications arise or medical therapy fails to control the disease [5,6]. Indications for surgery include strictures causing bowel obstruction, fistulas, abscesses, and perforations [7]. Despite advancements in medical therapies, up to 80% of patients with CD will require surgical resection during their lifetime [8]. However, surgery is not curative; the disease often recurs at or near the site of anastomosis.

The postoperative recurrence (POR) of CD remains a significant challenge in clinical practice. Endoscopic evidence of recurrence can be observed in up to 70% of patients within one year after ileocolonic resection, even in the absence of clinical symptoms [9]. Clinical recurrence typically follows endoscopic recurrence and can lead to complications necessitating further surgical interventions. Risk factors for POR include smoking, penetrating disease phenotype, prior intestinal surgery, and the presence of perianal disease [10]. Given the high incidence of POR in CD and the associated complications, there is a critical need for effective preventive and therapeutic strategies [11]. Emerging advanced biologic therapies, such as upadacitinib, risankizumab, and tofacitinib, have shown promise in managing moderate-to-severe CD [12]. This review aimed to evaluate the efficacy and safety of these agents in the context of POR, providing insights to inform clinical practice and guide future research.

## 2. Risk Factors Contributing to POR

**Figure 1:**
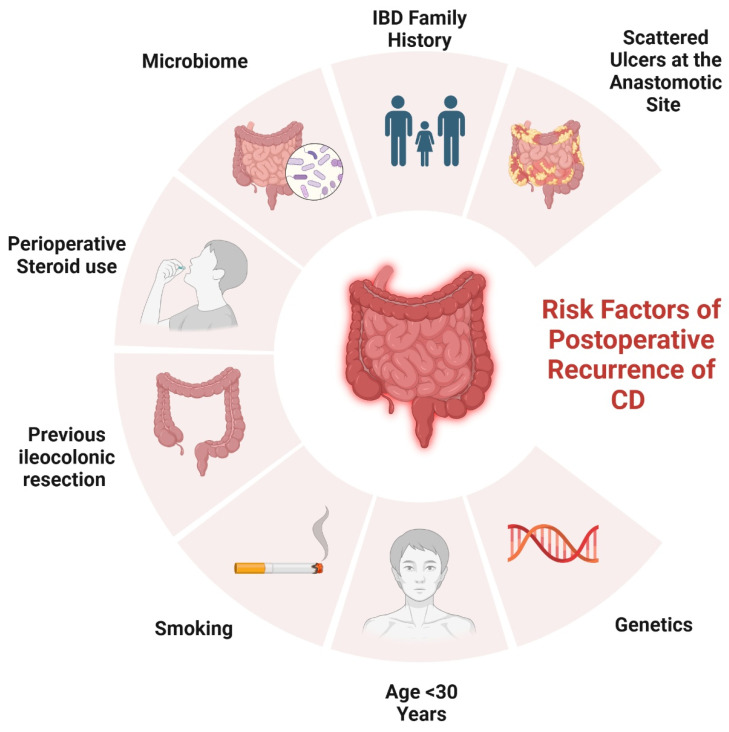
Risk factors of POR in CD.

Several risk factors have been identified that contribute to the likelihood of postoperative recurrence in CD (Figure 1).

### 2.1. Genetical Factors

Mutations in the NOD2/CARD15 gene have been linked to the development and progression of CD, particularly in predicting ileal stenotic disease and the need for early surgical intervention [13]. Among these, the 1007fs mutation has been associated with an increased risk of POR, with Renda et al. reporting a hazard ratio (HR) of 2.9 (95% CI 1.1–7.3, *p* = 0.03) [14]. However, conflicting results have been observed, as studies by Manconi et al. and Fowler et al. did not find a significant association [15,16]. Beyond NOD2, additional genetic loci have been evaluated. Individuals with reduced IL-10 production were more likely to experience endoscopic POR, though no specific IL-10 promoter polymorphism was identified [17]. The IRGFM polymorphism was correlated with frequent intestinal resections [18], while CARD8 mutations in homozygous individuals were linked to a higher risk of surgical recurrence (OR 7.56, 95% CI 1.13–50.37, *p* = 0.036) [19]. Moreover, SMAD3 gene mutations were found to elevate the likelihood of surgical recurrence (HR 4.04, 95% CI 1.77–9.21, *p* = 0.001) [15]. While these genetic markers may provide insight into CD prognosis, further studies are required to establish their predictive accuracy.

### 2.2. Surgical and Histological-Related Factors

Regarding the surgical approach, laparoscopic resection is preferred due to its advantages in recovery and cosmesis, but no significant difference in recurrence rates has been observed between laparoscopic and open surgery, as demonstrated in RCTs by Stocchi et al. [20] and Eshuis et al. [21], along with a meta-analysis by Patel et al. [22]. Strictureplasty, a bowel-sparing procedure, is a viable alternative to resection in select patients. However, recurrence rates vary depending on the technique. A meta-analysis by Tichansky et al. found that Heineke–Mikulicz (HM) strictureplasty was associated with higher recurrence rates compared to the Finney technique [23], while Reese et al. reported a non-significant increase in surgical recurrence for strictureplasty alone (38% vs. 31%, OR 1.36, 95% CI 0.96–1.93, *p* = 0.09) compared to resection [24]. Furthermore, blood transfusion has been investigated for its immunosuppressive effects, with recent data from a 318-patient cohort linking transfusion to increased risks of endoscopic recurrence (HR 2.08, 95% CI 1.38–3.14, *p* < 0.001) and surgical recurrence (HR 3.43, 95% CI 1.92–6.13, *p* < 0.001) [25]. Lastly, prior resections are a well-established risk factor for POR. A randomized trial by McLeod et al. found an OR of 1.78 (95% CI 1.06–2.90, *p* = 0.028) for endoscopic recurrence and OR of 2 (95% CI 1.14–3.60, *p* = 0.0016) for surgical recurrence in patients with prior resections [26].

The type of anastomosis plays a significant role in POR (Table 1). Studies suggest that side-to-side anastomosis (SSA), particularly the stapled SSA, may be associated with lower recurrence rates compared to end-to-end anastomosis (EEA) [27,28]. However, an RCT by McLeod et al. found no significant difference in recurrence rates between SSA and EEA, with endoscopic recurrence at 12 months reported as 42.5% for EEA and 37.9% for SSA (*p* = 0.55), and clinical recurrence at 21.9% and 22.7%, respectively (*p* = 0.92) [26]. Similarly, a meta-analysis by Simillis et al. (661 patients) found no significant difference between the two techniques in perioperative recurrence or reoperation rates [29]. A novel Kono-S anastomosis, designed to minimize ischemia and bacterial overgrowth, has shown low recurrence rates in preliminary studies, but further research is needed [30]. In terms of extent and resection margins, a landmark RCT by Fazio et al. demonstrated that limited resection margins (2 cm from the diseased bowel) were not associated with higher recurrence rates than wide resections (12 cm) [31]. While the length of resected bowel does not consistently predict recurrence, the European Crohn’s and Colitis Organisation (ECCO) guidelines identify extensive resection (>50 cm) as a predictive factor for POR [32]. These findings highlight the importance of surgical strategy in managing CD, emphasizing the need for individualized decision-making.

The role of granulomas in POR remains debated. Early studies suggested a protective effect, with Glass et al. associating granulomas with a lower POR risk [33], while Champers reported a better prognosis when granulomas were found in the large bowel and anus [34]. However, more recent research indicates an increased risk. A retrospective Mayo Clinic study (89 patients) found granulomas linked to a higher POR risk (HR 2.89, 95% CI 1.26–6.64, *p* = 0.01) [35]. A meta-analysis by Similis et al. (22 studies) concluded that granulomatous CD increases the risk of recurrence (OR 1.37, 95% CI 1.02–1.84, *p* = 0.04) and reoperation (OR 2.38, 95% CI 1.43–3.95, *p* < 0.001), leading ECCO guidelines to recognize granulomas as a POR risk factor [36]. Additionally, lymphatic vessel density has emerged as a potential biomarker. Rahier et al. found that decreased lymphatic density in the mucosa and submucosa at the proximal margin was associated with early POR, while a mucosal lymphatic density >7% predicted non-recurrence (sensitivity 81%, specificity 75%) [37]. Further research is needed to validate its prognostic value.

Plexitis has been widely studied for its potential role in predicting POR. A study by Ferrante et al. (59 patients) identified myenteric plexitis at the proximal resection margin as a significant predictor of early endoscopic recurrence at three months (OR 4.36, 95% CI 1.44–13.23, *p* = 0.008) and 12 months (OR 9.80, 95% CI 1.04–92.70, *p* = 0.041) [38]. However, submucosal plexitis did not show a significant correlation with POR. Subsequent studies provided mixed findings: Sokol et al. (171 patients) linked submucosal plexitis (≥3 mastocytes) with early clinical recurrence (HR 1.87, 95% CI 1.00–3.46, *p* = 0.048), while Bressenot et al. (67 patients) found an even higher risk when eosinophils (HR 8.02, 95% CI 1.87–34.47, *p* = 0.0054) or lymphocytes (HR 5.84, 95% CI 1.23–27.65, *p* = 0.0269) were present [39,40]. Another study (86 specimens) reported severe myenteric plexitis as a predictor for surgical recurrence (*p* = 0.035) [41]. Despite inconsistencies, French cohort studies and a Belgian prospective study confirmed myenteric plexitis as an independent risk factor for POR, particularly at the proximal margin [42,43].

### 2.3. Disease-Related Factors

The behavior of the disease significantly influences the POR risk. A penetrating phenotype (Montreal-B3) is associated with early POR, as shown in multiple studies, including Sachar et al., where none of the stricturing phenotype (Montreal-B2) patients experienced POR within three years [44]. Similarly, Avidan et al. (86 patients) found that penetrating disease significantly increased the POR risk and shortened the recurrence interval compared to smoking [45]. Two meta-analyses confirmed these findings: Similis et al. (13 studies, 3044 patients) reported that perforating disease increased the POR risk (HR 1.50, 95% CI 1.16–1.93, *p* = 0.002) [46], while Pascua et al. (12 studies) linked perforating disease to a higher risk of endoscopic POR (OR 1.59, 95% CI 1.37–1.84, *p* < 0.05) [47]. Consequently, the ECCO guidelines now include fistulizing disease as a POR risk factor [32]. Perianal disease is also widely accepted as a strong predictor of POR. A Korean study (132 patients) found that perianal lesions were an independent risk factor for reoperation (HR 1.98, 95% CI 1.03–3.78, *p* = 0.039) [48], while a population-based cohort (907 patients) demonstrated a relative risk of 1.6 (95% CI 1.2–2.3, *p* = 0.003) for POR [49]. Disease location also plays a role, with ileal involvement (Montreal-L1) being a predictive factor for a second ileocolonic resection (OR 2.42, 95% CI 1.02–5.78, *p* = 0.05) [50]. Similarly, a John Radcliffe Hospital study (280 patients) found that ileal disease was associated with higher POR rates compared to ileocolonic or colonic involvement [51], and a Netherlands population-based study supported the association between small bowel disease and POR [52]. While some studies link jejunal disease with increased surgical recurrence rates [53], the role of upper gastrointestinal involvement remains debated, with mixed findings from population-based studies [54].

### 2.4. Patient-Related Factors

Smoking is a well-established, modifiable risk factor for POR in CD. Cottone et al. identified smoking as a predictor for endoscopic recurrence (OR 2.2, 95% CI 1.2–3.8) and surgical recurrence (HR 2.0, 95% CI 1.2–2.3), while Unkart et al. found smokers had an increased risk of a second ileocolonic resection (HR 2.08, 95% CI 1.11–3.91, *p* = 0.023), as shown in Table 2 [55,56]. A meta-analysis of 16 studies (2692 patients) confirmed a 2-fold higher risk for clinical recurrence and a 2.5-fold increased risk for surgical recurrence in smokers at 10 years, while smoking cessation reduced the POR risk to that of non-smokers [57]. The dose–response effect has been documented, with heavy smokers (>15 cigarettes/day) having higher recurrence rates (77% vs. 45%, *p* = 0.02) [58]. However, some studies failed to confirm smoking as a risk factor, likely due to small sample sizes or missing smoking history data [50,59]. Sex has not been consistently associated with POR, with studies reporting conflicting results [60,61].

## 3. Classification and Monitoring of Postoperative Recurrence

POR can manifest in various forms, including endoscopic, clinical, radiologic, and surgical recurrence [62,63]. Endoscopic recurrence is typically the earliest detectable form and is identified through colonoscopy, revealing mucosal lesions at the anastomotic site or in the neoterminal ileum. Clinical recurrence involves the return of symptoms such as abdominal pain, diarrhea, and weight loss, which may or may not correlate with endoscopic findings [64]. Radiologic recurrence is identified through imaging modalities like magnetic resonance enterography or computed tomography enterography, showing structural changes indicative of active disease. Surgical recurrence refers to the necessity for additional surgical intervention due to complications or refractory disease.

Diagnosing the POR of CD requires a multifaceted approach incorporating clinical symptoms, biochemical markers in serum and feces, radiological imaging, and endoscopic evaluation [65]. However, differentiating POR from other postoperative complications, such as adhesional obstructions, dysmotility, or bile salt malabsorption-related diarrhea, can be challenging [66]. Endoscopy remains the gold standard for detecting POR, with the Rutgeerts’ score (RS) being the most widely used classification tool in clinical practice and trials [67,68]. The RS categorizes the severity of endoscopic lesions observed at the neoterminal ileum and ileocolonic anastomosis, which serve as predictors for future clinical activity [69]. Research has established that a higher RS score correlates with an increased risk of clinical recurrence over subsequent years, with a significant distinction observed between patients with minimal (i0–i1) versus moderate-to-severe (≥i2) lesions [70,71]. Furthermore, a systematic review by Ble et al. confirmed that patients with endoscopic recurrence (RS ≥ i2) face a markedly higher risk of clinical relapse, demonstrating a relative risk (RR) of 10.7 [64]. Lesions restricted to the anastomosis are categorized as i2a, while the presence of more than five aphthous ulcers or larger skip lesions falls under the i2b classification [72,73,74]. Recent studies have explored the potential differences in clinical outcomes between i2a and i2b lesions [75,76,77]; however, findings have been inconsistent, leaving uncertainty about whether these two subgroups should be managed and monitored with distinct treatment approaches. Guidelines recommend using the RS at six months post-surgery to guide CD management. Patients with i0–i1 lesions are at low risk of recurrence [78] and can continue current management with periodic noninvasive markers and repeat endoscopy in 1–3 years [79]. Conversely, i2, i3, or i4 lesions indicate early postoperative recurrence, warranting treatment escalation, often with biologic therapy [80].

Advances in surgical techniques, such as the Kono-S anastomosis, have demonstrated lower endoscopic recurrence rates compared to conventional anastomotic techniques, highlighting the impact of surgical approach on POR risk [81,82]. Given the variability in recurrence patterns, risk stratification tools incorporating surgical, anatomical, and clinical factors are needed [83]. The timing of postoperative ileocolonoscopy is crucial for detecting early recurrence and guiding therapeutic decisions, with six months being the optimal assessment point, as evidenced by the POCER trial [84,85]. Current guidelines recommend follow-up endoscopic evaluations within 6–12 months after surgery, with subsequent assessments guided by clinical and biomarker trends [86,87].

Cross-sectional imaging techniques, such as intestinal ultrasound (IUS) and magnetic resonance enterography (MRE), provide a comprehensive evaluation of the entire bowel wall, overcoming the limitation of endoscopy, which only assesses the mucosal surface [88]. These non-invasive modalities, free from ionizing radiation exposure, are increasingly favored for POR monitoring in CD. A meta-analysis by Yung et al. found that while capsule endoscopy had the highest sensitivity (100%) but a lower specificity (69%), MRE demonstrated a sensitivity of 97.3% and specificity of 83.7%, and IUS showed a sensitivity of 83.5% and specificity of 91.5% [89]. The MONITOR index, developed to assess POR through MRE, assigns scores based on bowel wall characteristics, with a cut-off of 1 yielding a sensitivity of 79% and specificity of 55% [90]. Despite its diagnostic utility, MRE’s high cost, limited availability, and contrast requirements restrict its widespread application.

IUS is emerging as a preferred alternative due to its accessibility and real-time imaging capabilities [91]. IUS findings correlate closely with endoscopic RS, and IUS has shown higher diagnostic yield than clinical indices or blood/stool markers in identifying early recurrence [92]. One study concluded that bowel ultrasound has “high diagnostic efficacy” for POR and can be considered a valid non-invasive alternative to ileocolonoscopy [91]. This noninvasive approach can allow earlier detection of subclinical recurrence and potentially reduce the need for routine surveillance colonoscopies [68]. IUS diagnostic accuracy can be enhanced through small intestine contrast ultrasound (SICUS) and contrast-enhanced ultrasonography (CEUS), which provide additional insights into bowel wall inflammation [93]. A meta-analysis by Rispo et al. reported that bowel wall thickness (BWT) > 3 mm on IUS had a sensitivity of 82% and specificity of 88% for detecting endoscopic recurrence, while SICUS demonstrated higher sensitivity (99%) but lower specificity (75%) [94]. A multicenter prospective study incorporating IUS and fecal calprotectin (FC) identified BWT per 1-mm increase, mesenteric lymph nodes, and FC ≥ 50 μg/g as independent predictors of endoscopic recurrence, correctly classifying 75% of patients [68]. An increased power Doppler signal (bowel wall hyperemia) is another hallmark of active disease; recurrent lesions often show marked hypervascularity on IUS, a feature that correlates significantly with endoscopic inflammation [91]. Other ultrasound signs of POR include the loss of normal bowel wall stratification and mesenteric changes such as creeping fat hypertrophy or enlarged lymph nodes [95]. Notably, the presence of prominent mesenteric lymph nodes on IUS was associated with a markedly higher risk of endoscopic recurrence (OR ≈ 15 in one prospective cohort) [68]. These findings underscore the potential role of IUS and FC in postoperative CD surveillance, potentially reducing the reliance on colonoscopy. However, further large-scale prospective studies are needed to validate these non-invasive monitoring strategies.

Biomarkers, particularly FC and serum markers, offer non-invasive alternatives for disease monitoring, aiding in risk stratification and optimizing endoscopic evaluation timing [96]. FC, a neutrophil-derived protein, strongly correlates with endoscopic and histologic inflammation [97]. A meta-analysis determined that an FC threshold of 150 μg/g had a sensitivity of 70% and specificity of 69% in predicting endoscopic recurrence (RS > i2), though methodological inconsistencies across studies limit its reliability [98]. Research by Boschetti et al. suggested that a lower threshold of 100 μg/g improved sensitivity (95%) but reduced specificity (54%), potentially decreasing the need for colonoscopy by 30% [99]. However, some studies have questioned the accuracy of FC in detecting POR, highlighting the importance of serial measurements over single assessments [100].

Serum biomarkers offer an additional approach to POR surveillance, with better patient adherence than stool-based assays [101]. However, standard inflammatory markers like C-reactive protein (CRP) lack sufficient sensitivity for early recurrence detection [101]. A promising advancement in this area is the Endoscopic Healing Index (EHI), a validated panel that quantifies 13 serum proteins involved in key pathways such as inflammation, angiogenesis, extracellular matrix remodeling, and immune regulation. These include angiopoietin 1 and 2, serum amyloid A1 (SAA1), interleukin-7 (IL-7), extracellular matrix metalloproteinase inducer (EMMPRIN), matrix metalloproteinases (MMP1, MMP2, MMP3, MMP9), transforming growth factor alpha (TGFA), carcinoembryonic antigen-related cell adhesion molecule 1 (CEACAM1), and vascular cell adhesion molecule 1 (VCAM1), in addition to CRP [102].

A validation study demonstrated that an EHI score < 20 had high sensitivity (83.2–97.1%) but variable specificity for identifying endoscopic remission, defined as a Simple Endoscopic Score for Crohn’s Disease (SES-CD) ≤ 2 [102]. Further analysis using data from the POCER trial found that at six months post-surgery, EHI and faecal calprotectin (FC) had comparable sensitivity (81.8% vs. 90.9%) and negative predictive values (84.0% vs. 91.7%) for detecting recurrence [103]. However, at 18 months, FC was a better predictor than EHI, with a negative predictive value of 89.7% compared to 64.9% [103]. While serum biomarkers such as the EHI show promise, FC remains the preferred non-invasive tool for monitoring POR, and further research is needed to validate serum-based markers as independent predictors of recurrence and establish their place in clinical practice.

## 4. Pathophysiology of POR

### 4.1. Pathophysiology

**Figure 2:**
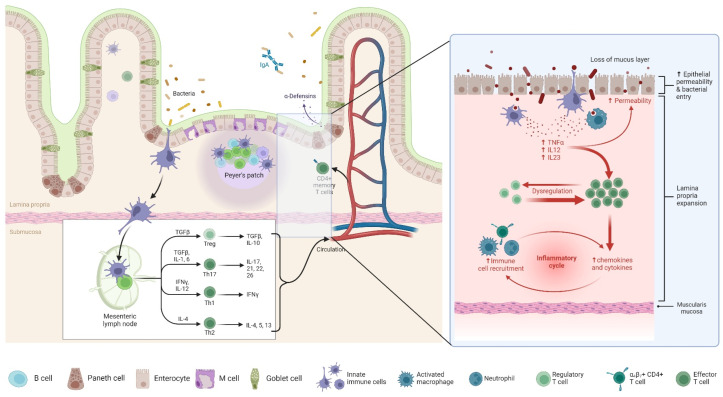
Pathophysiology of POR in CD. This figure illustrates the immune dysregulation underlying POR in CD. The left panel represents a normal intestinal mucosa with intact epithelial barriers, antimicrobial defenses (IgA and α-defensins), and organized immune surveillance via Peyer’s patches and mesenteric lymph nodes. Regulatory T cells (Tregs) maintain immune homeostasis by producing anti-inflammatory cytokines (TGF-β and IL-10). The right panel highlights the pathological mechanisms contributing to POR in CD. Loss of the mucus layer and increased epithelial permeability facilitate bacterial translocation, triggering an exaggerated immune response. Activated macrophages release pro-inflammatory cytokines (TNF-α, IL-12, and IL-23), leading to dysregulated immune activation, effector T cell expansion, and an inflammatory cycle characterized by enhanced recruitment of immune cells, upregulation of chemokines and cytokines, and lamina propria expansion. The chronic inflammatory response drives mucosal damage and fibrosis, increasing the risk of disease recurrence post-surgery.

The POR of CD is driven by a complex interplay of microbiological, immunological, and genetic factors [104]. The gut microbiota plays a pivotal role in disease recurrence, as demonstrated by studies linking dysbiosis to increased inflammation following ileocecal resection [105], as shown in Figure 2. Notably, Sokol et al. reported significant shifts in the ileal mucosa-associated microbiota, with an increased presence of Proteobacteria and a reduction in Firmicutes, correlating with endoscopic recurrence [106]. Additionally, alterations in luminal content flow at the anastomotic site, particularly in side-to-side anastomoses, facilitate bacterial proliferation and immune activation, further contributing to POR [107]. Genetic predisposition also influences recurrence risk, with the NOD2 gene playing a crucial role in microbial recognition and immune modulation. A meta-analysis highlighted that patients with NOD2 variants have a significantly higher likelihood of POR (odds ratio: 1.64, *p* = 0.003), and additional genes such as BACH2, CARD8, and TNFSF15 have been implicated in recurrence risk, though their mechanisms remain undefined [108]. Despite these findings, genetic testing is not yet recommended for routine clinical assessment due to the lack of standardized predictive biomarkers [109].

The immune system’s involvement in POR is also substantial, with both innate and adaptive immunity contributing to disease recurrence. Macrophages, serving as key mediators of inflammation, have been observed in increased numbers in the neoterminal ileum prior to endoscopic lesions, supporting their role in early POR pathogenesis [107]. Additionally, T-cell clonal expansion has been detected in resected bowel mucosa, suggesting that cell-mediated immunity plays a central role in recurrence, particularly among smokers [110]. Recent attention has also been given to the mesentery’s contribution to CD recurrence, with findings indicating that mesenteric excision may influence POR rates. The phenomenon of “creeping fat”, characterized by mesenteric adipose hypertrophy and immune cell infiltration, suggests that mesenteric lymph nodes may serve as reservoirs for CD immune memory, potentially facilitating disease recurrence [104]. However, evidence regarding the benefits of mesenteric excision remains inconclusive, with retrospective analyses yielding conflicting results, and current guidelines do not universally recommend routine mesenteric resection during ileocecal resection [111,112].

### 4.2. Mechanisms by Which Dysbiosis Contributes to Intestinal Inflammation

Dysbiosis, characterized by an imbalance in the gut microbiota, plays a pivotal role in the pathogenesis of IBD by disrupting mucosal homeostasis and promoting inflammation [113]. A notable consequence of dysbiosis is the reduction in beneficial commensal bacteria, such as *Faecalibacterium prausnitzii*, which are instrumental in producing short-chain fatty acids (SCFAs) like butyrate. Butyrate serves as a primary energy source for colonic epithelial cells and possesses anti-inflammatory properties by inhibiting nuclear factor kappa B (NF-κB) activation [114]. A diminished presence of these SCFA-producing bacteria leads to decreased butyrate levels, resulting in compromised epithelial barrier integrity and heightened susceptibility to inflammation [115].

Furthermore, dysbiosis often entails an overrepresentation of pathogenic bacteria, including certain Escherichia coli strains, which can exacerbate inflammatory responses. These pathogenic microbes may adhere to and invade epithelial cells, triggering the release of pro-inflammatory cytokines and recruiting immune cells to the intestinal mucosa [115]. Additionally, dysbiosis can lead to the overproduction of reactive oxygen species (ROS), causing oxidative stress that further damages the epithelial barrier and perpetuates the inflammatory cycle. This imbalance between protective and harmful microbial populations disrupts the delicate equilibrium of the gut environment, thereby contributing to the chronic inflammation characteristic of IBD [116].

## 5. Current Strategies and Guidelines of Prophylaxis

Ileocecal resection is not curative for CD, and a significant proportion of patients experience POR. The pivotal study by Rutgeerts et al. demonstrated that up to 73% of patients develop endoscopic lesions within one year post-surgery, a finding that has been confirmed in more recent studies [78,117]. However, the optimal strategy to prevent recurrence remains a subject of debate, as approximately 20–30% of patients do not develop endoscopic POR, while 40–50% exhibit only intermediate lesions (Rutgeerts i1–i2), which are associated with a lower risk of clinical and surgical recurrence [118]. Current management approaches include either systematic prophylactic therapy initiated early after surgery or an endoscopy-driven strategy in which treatment is started based on evidence of recurrence detected during a colonoscopy performed 6–12 months postoperatively [118]. International guidelines offer differing recommendations: the ECCO and the British Society of Gastroenterology (BSG) advocate prophylactic therapy for high-risk patients, while the American Gastroenterological Association (AGA) recommends prophylaxis for all patients, regardless of risk factors [32,86,119]. Furthermore, there is variation in defining high-risk patients, with the BSG requiring at least two risk factors, while ECCO and AGA consider a single risk factor sufficient [32,86,119].

Each strategy has its advantages and limitations. Systematic prophylaxis improves medium- to long-term remission rates but risks overtreatment, exposing patients to unnecessary drug-related side effects [118]. Conversely, an endoscopy-driven approach may prevent over- and undertreatment, but categorizing patients into high- and low-risk groups remains challenging. Studies have not definitively resolved this debate. Supporting the BSG guidelines, one study found that only patients with three or more risk factors had significantly higher odds of developing endoscopic POR [120]. In contrast, a retrospective analysis of 376 patients indicated that while prophylaxis reduced endoscopic POR at one year in high-risk patients (HR 0.48, *p* = 0.04), there was no significant difference in clinical recurrence within three years between the two strategies (HR 1.06, *p* = 0.82) [121]. Another study observed that in patients with only one risk factor, immediate prophylaxis did not significantly lower endoscopic or severe endoscopic POR rates within 12 months post-surgery [122]. However, a prospective cohort study of 213 CD patients who underwent ileocecal resection found that both low- and high-risk patients who did not receive prophylaxis had higher endoscopic POR rates compared to those who did (45% vs. 16%, *p* = 0.012; 49% vs. 26%, *p* = 0.019, respectively) [123]. Additionally, the PORCSE study found that the endoscopic POR rate was higher in the endoscopy-driven group compared to the early prophylaxis group (53.8% vs. 41.5%, *p* = 0.039), with significantly higher clinical POR (35.7% vs. 17.7%, *p* = 0.002) [124]. Similarly, a retrospective study reported that the absence of prophylactic therapy was independently associated with an increased endoscopic POR risk (OR 0.22; *p* = 0.0004) and a greater likelihood of hospitalization and surgery at five years [125].

## 6. Traditional Biological Therapies

Biologic therapies have revolutionized the management of CD, particularly for patients with moderate-to-severe forms unresponsive to conventional treatments. These therapies are derived from living organisms and specifically target components of the immune system to reduce inflammation. By inhibiting specific cytokines or their receptors, biologics can effectively diminish the inflammatory response characteristic of CD. The advent of advanced biologic agents has expanded treatment options, offering hope for improved disease control and quality of life for patients.

### 6.1. Anti-Tumor Necrosis Factor (TNF) Agents

Infliximab has shown efficacy in preventing POR in CD. A non-randomized study found infliximab with methotrexate superior to mesalamine (0% vs. 75% POR) [126], and retreatment restored remission in relapsed patients [127]. An RCT showed early infliximab use reduced recurrence at 12 and 36 months [128], while a pair-matched study identified it as the only factor preventing surgical recurrence (HR 0.08, *p* = 0.03) [129]. The PREVENT trial confirmed a significant reduction in endoscopic recurrence (30.6% vs. 60.0%, *p* < 0.001), though clinical recurrence differences were not significant (12.9% vs. 20.0%, *p* = 0.097) [130]. Adalimumab also reduced POR, with one study showing 75% of high-risk patients in remission at 24 months [131] and another reporting low clinical (13.7%) and endoscopic (20.7%) recurrence at one year [132]. However, to the best of our knowledge, there are no placebo-controlled RCTs exist for adalimumab. Comparative studies and meta-analyses found no significant difference between infliximab and adalimumab in preventing recurrence [133,134,135].

### 6.2. Anti-Integrin and Anti-Interleukin Agents

Data on the effectiveness of vedolizumab (VDZ) and ustekinumab (UST) in preventing POR of CD remain limited. Retrospective comparisons between vedolizumab and anti-TNF agents indicate a lower rate of ER at 6–12 months in vedolizumab-treated patients (25% vs. 66%, *p* = 0.01), though clinical and biological remission rates were comparable. Notably, vedolizumab was independently associated with a higher risk of endoscopic POR in both univariate and multivariate analyses [136]. Preliminary findings from the REPREVIO trial, a placebo-controlled RCT, demonstrated a significantly higher ER rate in patients receiving vedolizumab compared to placebo (42% vs. 3%, *p* < 0.001), with treated patients having a 77.8% probability of achieving a better RS than controls (*p* < 0.0001) [137]. In contrast, ustekinumab showed promise in a small-scale comparative study where patients receiving UST had a lower endoscopic POR rate at six months than those on azathioprine (28% vs. 54.5%, *p* = 0.029), though the difference was primarily driven by moderate disease (Rutgeerts i2) rather than severe recurrence (≥i3) [138].

Real-world evidence further supports the need for additional studies. A retrospective multicenter analysis of 297 patients receiving biologic prophylaxis for POR found that endoscopic recurrence at one year was 61.8% for UST, 33% for VDZ, and 40.2% for anti-TNF therapy. However, since patients treated with UST or VDZ had higher rates of prior surgery and biologic exposure, adjusted analyses showed a comparable risk of POR between groups (VDZ vs. anti-TNF: OR 0.55, 95% CI 0.25–1.19; UST vs. anti-TNF: OR 1.86, 95% CI 0.79–4.38) [139]. Another multicenter cohort study of 278 patients found that only early anti-TNF therapy (within four weeks) was associated with a reduced POR risk, while VDZ and UST did not show significant benefits (anti-TNF: aHR 0.61, 95% CI 0.40–0.93; VDZ: aHR 1.44, 95% CI 0.59–3.56; UST: aHR 2.06, 95% CI 0.84–5.06) [140]. A direct prospective comparison of vedolizumab vs. ustekinumab (40 UST-treated vs. 25 VDZ-treated) found similar clinical POR rates at 12 months (32% vs. 30%) and endoscopic POR rates at 18 months (42% vs. 40%) [141]. These findings highlight the need for further RCTs to clarify the role of ustekinumab and vedolizumab in POR prevention.

## 7. Advanced Biologic Therapies

### 7.1. Upadacitinib

The efficacy of upadacitinib in CD was established through two induction trials (U-EXCEL and U-EXCEED) and one maintenance trial (U-ENDURE) [142]. The induction studies included patients with moderate-to-severe CD, defined by frequent soft or liquid stools, an abdominal pain score of ≥2, and an SES-CD of ≥6. In U-EXCEL, 54.6% of patients had failed conventional therapy, while 45.4% had previously failed biologics. In contrast, U-EXCEED exclusively included biologic-experienced patients. The primary efficacy endpoints were clinical remission (CR) [Crohn’s Disease Activity Index (CDAI) < 150] and endoscopic response (>50% reduction in SES-CD) at week 12 for induction trials and week 52 for maintenance. Upadacitinib 45 mg daily demonstrated superior rates of CR and endoscopic response versus placebo in both induction studies, with some patients experiencing remission as early as weeks 2 and 4 [142]. In the U-ENDURE maintenance trial, patients who responded in the induction phase were randomized to receive 15 mg upadacitinib, 30 mg upadacitinib, or placebo. By week 52, both treatment arms achieved higher remission rates (15 mg: 37.3%, 30 mg: 47.6%, placebo: 15.1%) and greater ER (15 mg: 27.6%, 30 mg: 40.1%, placebo: 7.3%) compared to placebo [142]. These trials confirmed upadacitinib’s efficacy in inducing and maintaining remission in CD, with rapid onset of action and durable response rates.

In terms of real-world evidence, a multicenter retrospective cohort study assessed the effectiveness and safety of upadacitinib in IBD, across tertiary care centers. The study included 236 adult patients (≥18 years) who had received at least eight weeks of upadacitinib therapy. In 156 CD patients, steroid-free remission (76.8%, *p* < 0.001), CR (77.8%, *p* < 0.001), and clinical response (81.0%) were recorded at week 12, with a mean CDAI reduction from 214.9 to 117.5 (*p* < 0.001). ER was 19.4%, with a 48.9% endoscopic response and 4.9% mucosal healing. Radiological remission was achieved in 9.1%, with an 85.7% radiologic response, while intestinal ultrasound showed remission in 5.7% and response in 56.7% [143]. These findings highlight upadacitinib’s efficacy in achieving steroid-free remission, clinical response, and mucosal healing, particularly in biologic-resistant patients. Another multicenter retrospective study assessed the real-world effectiveness of selective JAK inhibitors in CD, focusing on bio-experienced patients; it included 246 patients, with 115 receiving upadacitinib for CD, and had a median follow-up of 7.5 months. At week 12, 76.2% of upadacitinib-treated CD patients achieved CR. By week 24, 76.9% achieved corticosteroid-free CR (CSFCR), while 50.0% had ER. At week 52, 66.7% remained in CSFCR, and 54.5% achieved ER. On the other hand, the study highlighted that upadacitinib was less effective in patients with stricturing and penetrating disease, as they were less likely to achieve CR by the end of induction (*p* = 0.04). Significant reductions in CRP (*p* < 0.0001) and fecal calprotectin (*p* < 0.0001) were observed as early as week 2, indicating a rapid anti-inflammatory effect [144]. These findings suggest that selective JAK1 inhibition with upadacitinib is an effective and well-tolerated treatment option for refractory moderate-to-severe CD, particularly in patients without penetrating or stricturing complications.

Moreover, a retrospective multicenter study assessed the real-world persistence, effectiveness, and safety of upadacitinib in IBD patients, with a minimum follow-up of 12 weeks. The study included 100 patients, with 68 diagnosed with CD. Participants had previously received a median of four advanced therapies. CR was achieved in 59% at week 8, 64% at week 12, and 42% at week 52. Dose escalation successfully restored remission in 60% of patients experiencing relapse. Additionally, 80% of patients with active immune-mediated diseases or extraintestinal manifestations showed improvement with upadacitinib therapy [145].

In patients with difficult-to-treat CD, a multicenter observational cohort study included 64 patients with moderate-to-severe CD (Harvey-Bradshaw Index [HBI] > 8 or SES-CD > 6), all of whom had failed corticosteroids, thiopurines, infliximab, adalimumab, vedolizumab, and ustekinumab. Many had prior surgical interventions (67.2%) and extra-intestinal manifestations (43.8%), with the majority having ileocolonic disease (59.4%). Patients received upadacitinib 45 mg/day for 12 weeks (induction) followed by 30 mg/day for 12 weeks (maintenance). The primary endpoint, CSFCR (HBI ≤ 3) at 12 weeks, was achieved in 33 patients (51.6%), while clinical response (HBI reduction > 3 points) was observed in 46 patients (71.9%). Biochemical remission (FC < 150 μg/g and CRP < 0.5 mg/dL) was documented in 36.2% of patients, while deep remission (clinical + biochemical remission + transmural healing) occurred in 21.8%. Ultrasonographic assessments at week 12 showed transmural healing in 28.8% of patients and transmural response in 73%. At week 24, among the 50 patients who continued upadacitinib, CR was sustained in 39 patients (78%), while clinical response persisted in 48 patients (96%). Biochemical remission was achieved in 38.3% [146].

Regarding refractory CD in pediatrics, a multicenter retrospective study evaluated the effectiveness and safety of upadacitinib as an induction therapy in pediatric CD across 30 international centers. The study included 100 children (median age: 15.8 years), all previously exposed to at least one biologic, with 89% having failed two or more biologic therapies. After eight weeks of induction therapy, 75% achieved a clinical response, 56% reached CR, and 52% achieved corticosteroid- and exclusive enteral nutrition-free CR. Additionally, 68% showed normalization of CRP, and 58% had FC < 150 mcg/g, with 13% achieving both enteral nutrition-free CR and FC remission [147]. Another single-center retrospective cohort study evaluated the real-world efficacy and safety of upadacitinib in pediatric IBD patients (ages 9–20 years). The study included 20 children and adolescents diagnosed with CD (n = 3), UC (n = 13), and IBD-unclassified (IBD-U) (n = 4). The primary outcome was clinical response, defined as a ≥20-point reduction in the Pediatric Ulcerative Colitis Activity Index (PUCAI) or ≥12.5-point decrease in the Pediatric CDAI (PCDAI). At week 8 (UC/IBD-U) and week 12 (CD), 90% of patients achieved clinical response, while 75% reached SF-CR post-induction, with 65% maintaining remission at week 24. PUCAI scores significantly improved in UC/IBD-U patients, and FC levels showed a downward trend post-induction, though CRP levels remained unchanged. Endoscopic response was observed in seven out of eight evaluated cases, with three achieving ER. Two patients discontinued therapy due to surgical interventions (subtotal colectomy and ileostomy for rectal perforation), but no new safety concerns were identified [148]. These findings indicate that upadacitinib is a promising therapeutic option for pediatric IBD patients refractory to approved treatments, with encouraging efficacy and a manageable safety profile.

While there is currently no direct evidence evaluating the efficacy of upadacitinib in preventing POR of CD, its proven effectiveness in inducing and maintaining remission in moderate-to-severe and refractory CD suggests a potential role in this setting. Clinical trials such as U-EXCEL, U-EXCEED, and U-ENDURE have demonstrated significant clinical and ER rates, even in patients with prior treatment failures. Moreover, real-world studies have confirmed upadacitinib’s effectiveness in biologic-experienced and highly refractory CD patients, with notable improvements in CSFCR, mucosal healing, and inflammatory markers. In difficult-to-treat CD, upadacitinib has shown promising clinical and biochemical remission rates, as well as transmural healing, which is a crucial marker of long-term disease control. Furthermore, its efficacy in pediatric CD—a population with limited treatment options—reinforces its potential as a viable alternative for patients at high risk of recurrence. Given that POR is driven by persistent inflammation, immune dysregulation, and mucosal barrier dysfunction, the selective JAK1 inhibition offered by upadacitinib may provide an anti-inflammatory effect capable of modifying disease recurrence patterns. Future randomized controlled trials and prospective cohort studies are needed to assess whether upadacitinib could be a viable strategy to prevent POR, particularly in high-risk patients, and to determine its role compared to conventional prophylactic therapies such as thiopurines, anti-TNFs, and IL-12/23 inhibitors.

### 7.2. Risankizumab

A phase II randomized, placebo-controlled trial evaluated risankizumab for moderate-to-severe CD, enrolling 121 patients, 93% of whom had previously failed TNF-antagonists or vedolizumab. Patients received intravenous risankizumab (600 mg or 200 mg) or placebo at weeks 0, 4, and 8, with CR (CDAI < 150) achieved in 24% (200 mg), 37% (600 mg), and 15.4% (placebo, *p* = 0.0489). Secondary endpoints, including clinical response (31% vs. 15%, *p* = 0.0489), ER (17% vs. 3%, *p* = 0.0015), and deep remission (7% vs. 0%, *p* = 0.0107), were significantly improved in the risankizumab groups [149]. In an open-label extension study, patients who did not achieve CR and ER at week 12 received risankizumab 600 mg IV every four weeks for an additional 12 weeks, while those in deep remission underwent a 12-week washout period. By week 26, CR rates were 55% in the placebo group, 59% in the 200 mg risankizumab group, and 47% in the 600 mg group. Among 62 patients who maintained CR at week 26, maintenance therapy with 180 mg subcutaneous (SC) risankizumab was initiated, resulting in 71% sustaining CR, 35% achieving ER, and 29% reaching deep remission at week 52 [150]. In a subsequent open-label extension, 65 patients continued risankizumab for a median of 33 months, with over 71% maintaining CR and more than 42% achieving ER by week 112, highlighting its durable long-term efficacy [151].

In 2022, the ADVANCE trial evaluated induction therapy for moderate-to-severe CD in patients with prior biologic or conventional therapy failure [152]. A total of 850 patients were randomized (2:2:1) to receive IV risankizumab (600 mg or 1200 mg) or placebo at weeks 0, 4, and 8. The co-primary endpoints at week 12 were CR (CDAI < 150 in the USA; stool frequency ≤ 2.8 and abdominal pain score ≤ 1 in non-USA countries) and endoscopic response (>50% reduction in SES-CD or ≥2-point decrease for isolated ileal disease with baseline SES-CD of 4). Risankizumab outperformed placebo across all primary endpoints. CR was achieved in 45% (600 mg, *p* < 0.0001) and 42% (1200 mg, *p* < 0.0001) vs. 25% (placebo), while stool frequency and abdominal pain-based remission rates were 43% (600 mg, *p* < 0.0001) and 41% (1200 mg, *p* < 0.0001) vs. 22% (placebo). Additionally, 40% (600 mg, *p* < 0.0001) and 32% (1200 mg, *p* < 0.0001) of risankizumab-treated patients achieved endoscopic response, compared to 12% on placebo [152].

The MOTIVATE induction trial was a multicenter, randomized, double-masked study that evaluated clinical and ER in patients with CD who had previously failed biologic therapy. A total of 569 patients were randomized 1:1:1 to receive risankizumab 600 mg, 1200 mg, or placebo every four weeks. At week 12, CR rates based on CDAI (<150) were 42% (600 mg, *p* < 0.0001) and 40% (1200 mg, *p* < 0.0001), significantly higher than the 20% in the placebo group. Similarly, remission based on stool frequency and abdominal pain scores was 35% (600 mg, *p* = 0.0007) and 40% (1200 mg, *p* < 0.0001) versus 19% for placebo. Endoscopic response rates at week 12 were also superior in the risankizumab groups (29% and 34%, *p* < 0.0001) compared to the placebo group (11%). No significant differences in efficacy were observed between the 600 mg and 1200 mg doses in either the ADVANCE or MOTIVATE trials [153].

The FORTIFY trial enrolled patients who achieved CR in the ADVANCE or MOTIVATE trials. Participants were randomized to receive SC risankizumab (180 mg or 360 mg) or placebo every eight weeks. At week 52, CR rates were significantly higher in the 180 mg (55%, *p* = 0.0031) and 360 mg (52%, *p* = 0.0054) groups compared to placebo (41%). Endoscopic response was also superior in both risankizumab groups (47%, *p* < 0.0001) vs. the placebo group (22%). Mucosal healing at week 52 occurred in 31% of patients receiving 360 mg risankizumab SC vs. 10% in the placebo group (*p* < 0.001), while ER was achieved in 39% of risankizumab-treated patients compared to 13% on placebo (*p* < 0.001) [154].

Both the ADVANCE and MOTIVATE trials showed that patients receiving risankizumab had greater rates of mucosal healing and ER. In ADVANCE, mucosal healing was achieved at week 12 by 21% of patients receiving risankizumab (600 mg) versus 8% in the placebo group, while in MOTIVATE, the rates were 14% vs. 4% for placebo. Similarly, ER was observed in 24% (ADVANCE) and 19% (MOTIVATE) of patients receiving risankizumab, compared to 9% and 4% in the placebo groups, respectively [154].

A direct comparison between ustekinumab and risankizumab in biologic-experienced CD patients was conducted in the SEQUENCE study [155]. The primary endpoint (CDAI-based CR at week 24) demonstrated the non-inferiority of risankizumab to Ustekinumab, with remission rates of 59% vs. 40%, respectively. For the second primary endpoint (ER at week 48, SES-CD ≤ 4 with a ≥2-point reduction and no subscore > 1), risankizumab was significantly superior to ustekinumab (32% vs. 16%, *p* < 0.0001) [156].

Although no direct studies have evaluated the use of risankizumab for the prevention of POR in CD, its demonstrated efficacy in induction and maintenance trials suggests a potential role in this setting. The consistent achievement of CR, endoscopic response, and mucosal healing in both biologic-naïve and biologic-experienced patients highlights its robust anti-inflammatory effects, which are crucial in preventing disease recurrence following surgical resection. Furthermore, the SEQUENCE trial’s findings of risankizumab’s superior endoscopic outcomes compared to ustekinumab further support its potential utility in maintaining post-operative remission. Given that preventing POR requires effective suppression of residual inflammation, risankizumab’s ability to achieve deep and durable remission could make it a valuable option for patients at high risk of recurrence. Future clinical trials specifically investigating its role in POR are warranted to determine its efficacy in this indication and to establish optimal treatment strategies.

## 8. Barriers to Implementing Advanced Biologic Therapies in Preventing Post-Operative Recurrence of Crohn’s Disease

Implementing advanced biologic therapies for the prevention of POR in CD faces several significant barriers, including high treatment costs, limited accessibility, and disparities in healthcare infrastructure. The annual direct medical costs for CD are substantial, with estimates around USD 4466 per patient, and these costs escalate with the use of biologic therapies and hospitalizations [157,158]. For instance, a study reported that patients experiencing endoscopic recurrence incurred median healthcare costs of USD 26,347, compared to USD 2729 for those in remission, highlighting the financial burden associated with disease management [157]. Additionally, the availability of biologic agents varies globally, influenced by differences in healthcare policies, insurance coverage, and economic resources. These disparities can lead to inconsistent treatment approaches and may limit the timely initiation of effective therapies. Addressing these challenges requires comprehensive strategies, including policy reforms to improve insurance coverage, initiatives to reduce medication costs, and investments in healthcare infrastructure to ensure equitable access to advanced treatments for all CD patients.

## 9. Future Directions and Research Opportunities

### 9.1. Ongoing Clinical Trials and Emerging Therapies

Despite the significant progress in biologic therapy for CD, the prevention and management of POR remain major clinical challenges. Ongoing clinical trials are investigating novel therapeutic strategies to optimize POR management, including emerging biologics and small molecules with different mechanisms of action. IL-23 inhibitors, such as risankizumab and mirikizumab, are under evaluation for their role in reducing post-operative inflammation and recurrence. The potential of JAK inhibitors, including upadacitinib, in preventing POR is also an area of active research, given their rapid onset of action and promising efficacy in biologic-experienced patients with CD. Combination therapy approaches are another avenue being explored to enhance the durability of remission post-surgery. Trials assessing the concurrent use of anti-TNF agents with IL-23 inhibitors or JAK inhibitors may provide insights into superior mucosal healing and THE sustained suppression of inflammation. Additionally, controlled-release formulations of immunomodulators and biologics, designed for localized intestinal targeting, are being developed to improve drug efficacy while minimizing systemic side effects.

### 9.2. Personalized Medicine Approaches in Biologic Therapy Selection for POR

The selection of biologic therapy for preventing POR remains largely empirical, often based on clinical risk stratification. However, personalized medicine approaches are emerging as critical tools for optimizing post-operative treatment strategies. Stratifying patients based on disease phenotype, previous treatment response, and genetic or immunologic markers may help tailor biologic selection to maximize efficacy. Therapeutic drug monitoring (TDM) has shown promise in optimizing the use of anti-TNF therapy in preventing POR, ensuring adequate drug levels while mitigating the risk of immunogenicity. Additionally, multi-omic profiling, including transcriptomics and microbiome analysis, is being investigated to identify specific patient subgroups that may benefit from alternative biologic pathways, such as IL-23 or JAK-STAT inhibition. The integration of artificial intelligence and machine learning models into clinical decision making could further enhance individualized treatment selection, improving long-term outcomes in post-operative patients.

Shared decision making (SDM) empowers patients to collaborate with healthcare providers in selecting treatment options that align with their preferences, values, and lifestyle, thereby improving adherence and satisfaction [159]. Implementing SDM in POR management involves comprehensive patient education about the risks and benefits of various prophylactic therapies, including biologic agents. This approach ensures that patients are well informed about potential complications, such as infections or injection site reactions, and the necessity for regular monitoring [160]. Moreover, discussing the implications of different treatment pathways allows patients to weigh the impact on their quality of life and make choices that best suit their individual circumstances.

Bioethical considerations are integral to personalized medicine in this setting. Ensuring equitable access to advanced therapies is paramount, as disparities in healthcare infrastructure and socioeconomic factors can limit availability [161]. Healthcare providers must advocate for policies that promote fairness and address these inequities. Additionally, maintaining patient confidentiality and data security is essential, particularly when utilizing genetic or biomarker information to guide treatment decisions. Obtaining informed consent and safeguarding personal health information are critical to upholding ethical standards and patient trust.

### 9.3. Potential Biomarkers for Predicting Response to Biologics in POR

The identification of reliable biomarkers for predicting biologic response in POR is an essential area of research. Traditional inflammatory markers such as FC and CRP have limited specificity in post-operative settings, necessitating the exploration of novel predictive biomarkers. Several emerging biomarkers, including oncostatin M (OSM), serum IL-23 levels, and gut-specific transcriptomic signatures, are under investigation for their potential to guide biologic selection in preventing POR. Endoscopic and histologic markers, such as the RS and the EHI, are being refined to serve as early indicators of biologic efficacy in post-operative patients. The use of non-invasive imaging modalities, including intestinal ultrasound and MRI-based inflammation markers, may further enhance the early detection of POR and allow timely therapeutic adjustments.

## 10. Conclusions

Despite advances in biologic therapies, the prevention and management of POR in CD remain challenging. While anti-TNF agents, such as infliximab and adalimumab, have been the cornerstone of POR prevention, newer biologics—including IL-23 inhibitors and JAK inhibitors—are emerging as promising alternatives, particularly for biologic-experienced patients. However, direct evidence supporting their use in POR is still limited, highlighting the need for well-designed clinical trials to evaluate their efficacy in the post-operative setting.

Future research should focus on identifying optimal biologic sequencing strategies, evaluating combination therapy approaches, and refining predictive models for patient stratification. The integration of TDM and biomarker-driven treatment selection may improve biologic persistence and efficacy in preventing POR. Additionally, real-world studies assessing the long-term outcomes of IL-23 and JAK inhibitors in preventing surgical recurrence are needed to guide clinical practice. As the landscape of biologic therapy continues to evolve, a more personalized and evidence-based approach to POR management will be essential in improving patient outcomes and reducing the need for repeat surgery.

## Figures and Tables

**Table 1:** Surgical techniques and POR.

Surgical Technique	Pros	Cons
**End-to-end anastomosis**	Standard technique; broadly familiar to surgeons	Higher risk of endoscopic recurrence compared to side-to-side
**Side-to-side anastomosis**	Lower post-operative recurrence rate; wider lumen reduces stricture risk	May be technically more demanding in certain anatomical settings
**Kono-S anastomosis**	Significantly lowers endoscopic and clinical recurrence rates; preserves mesentery	Requires specific surgical expertise and longer operative time
**Strictureplasty**	Bowel-preserving; recurrence usually occurs at other sites, not at the strictureplasty	Not suitable for perforating disease or when cancer is suspected
**Mesenteric excision (aggressive)**	May significantly reduce recurrence by removing mesenteric inflammatory drive	More extensive surgery; increased risk of complications

**Table 2:** Summary of risk factors for post-operative recurrence in Crohn’s disease.

Risk Factor Category	Specific Risk Factor	OR/HR (95% CI)	Reference
**Genetic**	NOD2 1007fs mutation	HR 2.9 (1.1–7.3)	Renda et al. [14]
	CARD8 homozygous	OR 7.56 (1.13–50.37)	Germain et al. [19]
	SMAD3 gene mutations	HR 4.04 (1.77–9.21)	Fowler et al. [15]
**Surgical**	Prior resections	OR 1.78 (1.06–2.90)	McLeod et al. [26]
	Blood transfusion (endoscopic)	HR 2.08 (1.38–3.14)	Li et al. [25]
	Blood transfusion (surgical)	HR 3.43 (1.92–6.13)	Li et al. [25]
**Histological**	Granulomas	HR 2.89 (1.26–6.64)	Malireddy et al. [35]
	Myenteric plexitis	OR 9.80 (1.04–92.70)	Ferrante et al. [38]
	Submucosal plexitis with eosinophils	HR 8.02 (1.87–34.47)	Bressenot et al. [40]
**Disease-related**	Penetrating disease	HR 1.50 (1.16–1.93)	Simillis et al. [46]
	Perianal disease	HR 1.98 (1.03–3.78)	Han et al. [48]
	Ileal involvement	OR 2.42 (1.02–5.78)	Pascua et al. [50]
**Patient-related**	Smoking (endoscopic recurrence)	OR 2.2 (1.2–3.8)	Cottone et al. [55]
	Smoking (surgical recurrence)	HR 2.0 (1.2–2.3)	Cottone et al. [55]

HR: hazard ratio, OR: odds ratio, CI: confidence interval.
